# Mixed oxides with corundum-type structure obtained from recycling can seals as paint pigments: color stability

**DOI:** 10.3762/bjnano.14.37

**Published:** 2023-04-05

**Authors:** Dienifer F L Horsth, Julia de O Primo, Nayara Balaba, Fauze J Anaissi, Carla Bittencourt

**Affiliations:** 1 Chimie des Interactions Plasma-Surface (ChIPS), Research Institute for Materials Science and Engineering, University of Mons, 7000 Mons, Belgium; 2 Chemistry Departament, Universidade Estadual do Centro-Oeste, Guarapuava, 85040-167, Brazil

**Keywords:** circular economy, colorimetry, sustainability

## Abstract

Green chromium and red iron oxides are technically important pigments due to their high color intensity, good dispersibility in paints, and superior hiding power. We report on the synthesis of colored pigments of mixed oxides with a corundum-type structure. The pigments are obtained via the addition of coloring ions to boehmite from recycled metallic aluminium. X-ray diffractometry (XRD) and Raman spectroscopy confirmed the crystallographic phase. Additionally, the oxidation state 3+ responsible for the greenish (chromium) and reddish (iron) coloration of the mixed oxides was confirmed by XPS and visible-light absorption measurements. The colorimetric stability of the oxides in acid and alkaline environments was evaluated. After 240 h of exposure to harsh environments, both pigments demonstrated stability and showed no strong color difference.

## Introduction

The growing interest in industrial products that do not harm the environment triggered the development of diverse strategies to optimize recycling and green syntheses of materials. It is possible to combine economic and environmental interests to produce synthetic inorganic pigments [1] using metallic aluminium scrap as precursor to obtain a white matrix that can then be colored by chromophore ions as an approach within the circular economy of aluminium [1]. Aluminium production has one of the most significant energy consumption differences between the primary (bauxite extraction) and secondary (recycling) synthesis routes. The energy consumption for obtaining secondary aluminium is reduced by 95%, as its raw source is aluminium scrap and used metallic aluminium (i.e., sheets, extrusion, turning, can seals) [2]. In addition, there is a reduction to 5% in greenhouse gas emissions compared to the direct synthesis, reducing the environmental impact. Besides, one ton of recycled aluminium saves up to eight metric tons of bauxite [3–4]. The amount of recycled aluminium packaging depends on individual national recycling policies, with rates ranging from 25% to 85% worldwide [3].

In the last decade, interest in the circular economy issues has increased almost a hundredfold [5], accompanied by research and widespread awareness. However, circular economy is still a relatively new concept. It has been embraced as a concept based on reducing, repairing, recycling, remanufacturing, and redirecting the life cycle of materials [6], aiming at a regenerative economy without waste [5]. Therefore, this study’s purpose is to prepare synthetic inorganic pigments starting from a synthetic route that includes aluminium recycling from can seals as a form of circular economy, where recycled aluminium becomes part of an industrial scope different from the initial one.

Inorganic pigments are widely sought after for their colors [7]. The color of a pigment is affected by the size and shape of the material’s particles [8]. However, the color assignment is based on the intrinsic light absorption characteristics of the pigment due to the presence of a chromophore ion [8]. Different transition metals such as V, Cr, Mn, Fe, Co, Ni, and Cu have been used for this purpose [9]. Using inorganic compounds in the synthesis tends to increase the chemical and physical stability of the pigments, ensuring greater durability. Furthermore, it reduces environmental problems because it avoids consuming natural raw materials, saving primary resources [10–11]. Inorganic pigments, generally, are less affected by light, ambient temperature, chemicals, and harsh atmospheres than organic pigments [12]. In addition, inorganic pigments offer the advantage of lower production cost [12] when using recycling materials as a precursor.

Global demand for pigments was around 12 million tons in 2020 and is dominated by titanium dioxide white pigment [11]. However, iron oxide red and chromium oxide green are technically important inorganic pigments due to color intensity, good dispersibility in paints, and superior hiding power [11]. Functionally, green is widely used as approval, while red is associated with prohibition and warning. The rationalization that red is associated with hazard makes it directly correlate with danger words or symbols (e.g., in chemical industry hazard symbols). In this way, green is the perfect contrast to red, as they are complementary colors in well-established chromatic models. Thus, green takes on the opposite meaning of danger, namely safety [13]. Therefore, it is essential to have color intensity and stability to avoid aging in signalization, for example, in motor roads, chemical industries, and hospitals.

Chromium oxide green belongs to the class of inorganic green and blue-green pigments [14]. It exhibits the corundum structure and a color change with increasing particle size from brighter yellow-green to darker blue-green [14]. Usually, chromium oxide pigments are synthesized starting from dichromats with chromium in the oxidation state +6, which are toxic [15]. However, using boehmite as a host matrix, the opportunity arises to use chromium as a coloring ion to obtain the same color result, without using toxic precursors. Remarkably, iron oxide red belongs to the class of yellow, red, and brown ocher, whose color depends on the crystallographic phase [11]. The hematite phase (α-Fe_2_O_3_) is the most stable, and its shade and color can be adjusted by the calcination temperature or the combination with other metals as coloring ions [11]. In this context, applying both Cr and Fe ions as coloring ions in a white matrix such as aluminium oxide is interesting.

We report on the synthesis of synthetic inorganic pigments using boehmite (γ-AlO(OH)) obtained from recycling aluminium can seals as a precursor. The boehmite phase was chosen because it is a lamellar phase and can allocate ions between the lamellas. The aluminium recycling process to obtain boehmite is based on the acid digestion of metallic aluminium can seals. After obtaining boehmite, we added chromium and iron ions to obtain colored mixed oxides with a corundum-type structure. The stability of the synthesized pigments in acid and alkaline environments was evaluated by colorimetric measurements.

## Results and Discussion

### X-ray diffractometry (XRD)

**Figure 1:**
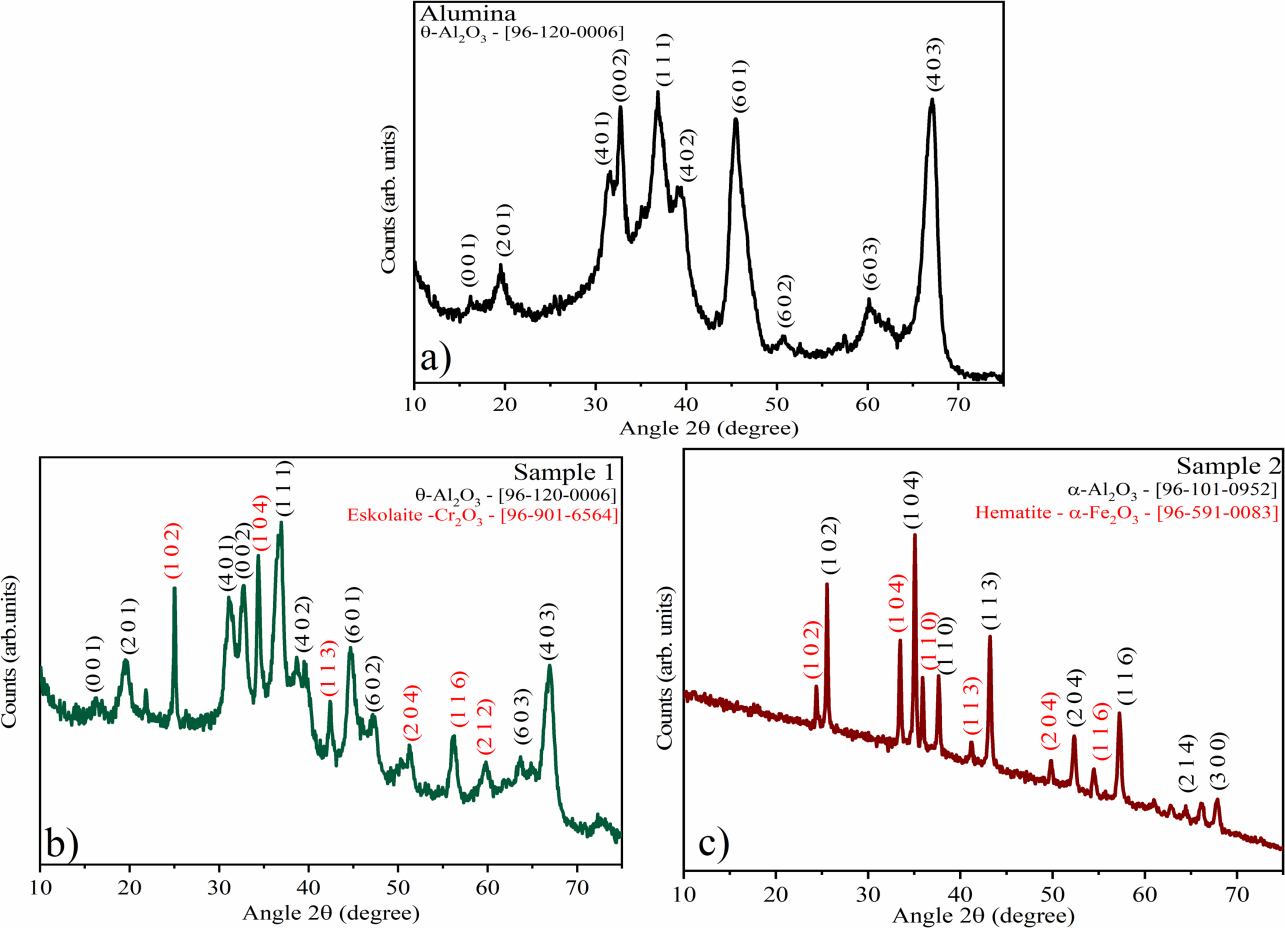
XRD diffractograms of (a) alumina showing the θ-Al_2_O_3_ phase, (b) sample 1 showing a mixture of alumina and eskolaite phases Cr_2_O_3_, and (c) sample 2 showing a mixture of alumina and hematite phases (α-Fe_2_O_3_).

The XRD of the pristine sample (alumina) (Figure 1a) shows single-phase θ-Al_2_O_3_, which is an alumina phase obtained through heat treatment above 900 °C [16]. In the diffractogram of sample 1 (Figure 1b), two phases are observed, θ-Al_2_O_3_ [96-120-0006] and eskolaite α-Cr_2_O_3_ [96-901-6564]. The crystal structure of the eskolaite phase is that of corundum (Al_2_O_3_), based on a hexagonal matrix of oxygen with two thirds of the octahedral sites filled with Cr^3+^ ions [17]. The phase mixture observed in sample 2 (Figure 1c) consists of α-Al_2_O_3_ [96-900-8082] and hematite (α-Fe_2_O_3_) [96-591-0083]. The indexing of the hematite phase indicates a rhombohedral structure [18]. The same crystallographic charts were indexed for materials with 5 and 20 wt % of coloring ions (Figure S1, Supporting Information File 1). The crystallinity of the samples was calculated considering the entire diffractogram. It is similar among the oxides, ranging from 57.3% (alumina) to 63.9% (sample 2) (Table 1). The crystallinity of the synthesized oxides is superior to the ones obtained via coprecipitation [1].

**Table 1:** Phase, crystallographic chart and crystallinity of alumina, sample 1, and sample 2.

Sample	Phase	Chart	Crystallinity (%)

alumina	θ-Al_2_O_3_	[96-120-0006]	63.9
sample 1	θ-Al_2_O_3_ α-Cr_2_O_3_	[96-120-0006] [96-901-6564]	57.3
sample 2	α-Al_2_O_3_ α-Fe_2_O_3_	[96-900-8082] [96-591-0083]	62.4

### Raman spectroscopy

**Figure 2:**
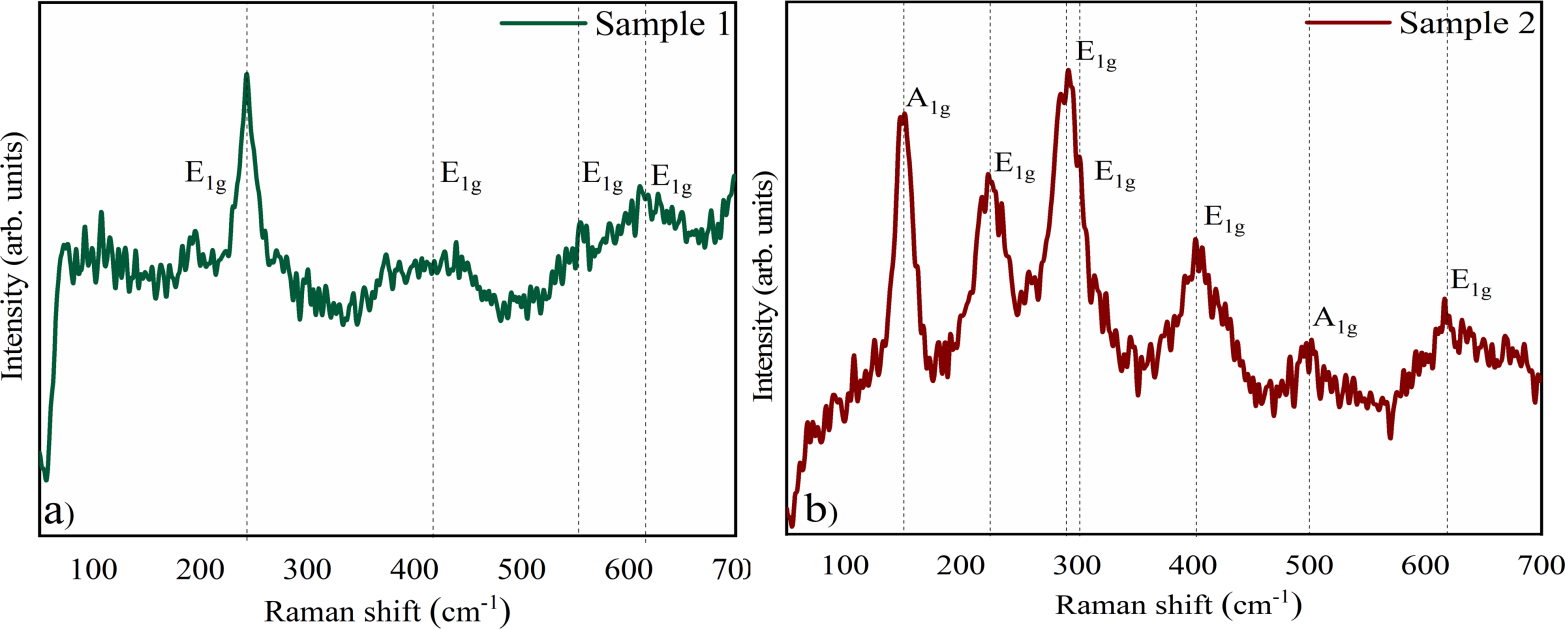
Raman spectroscopy of (a) sample 1, which presents the four vibrational modes *E*_1_*_g_* characteristic of Cr_2_O_3_, and (b) sample 2, which features the seven characteristic vibrational modes (two *A*_1_*_g_* modes and five *E*_1_*_g_* modes) of Fe_2_O_3_.

The Raman spectrum observed for sample 1 (Figure 2a) is characteristic of chromium oxide (Cr_2_O_3_), in agreement with what was observed by XRD. The spectrum is composed of four *E*_1_*_g_* vibrational modes (ca. 242 cm^−1^, ca. 413 cm^−1^, ca. 525 cm^−1^, and ca. 605 cm^−1^), as previously reported [19]. The Raman spectrum of sample 2 (Figure 2b) presents the seven optical symmetry modes expected for hematite (α-Fe_2_O_3_) in agreement with the XRD results. The Raman modes are *A*_1_*_g_* (ca. 149 cm^−1^ and ca. 501 cm^−1^) and *E*_1_*_g_* (ca. 222 cm^−1^, ca. 290 cm^−1^, ca. 298 cm^−1^, ca. 402 cm^−1^, and ca. 615 cm^−1^, where 290 cm^−1^ and 298 cm^−1^ usually are a doublet with *E*_1_*_g_* symmetry and cannot be easily resolved [20].

### Scanning electron microscopy (SEM)

**Figure 3:**
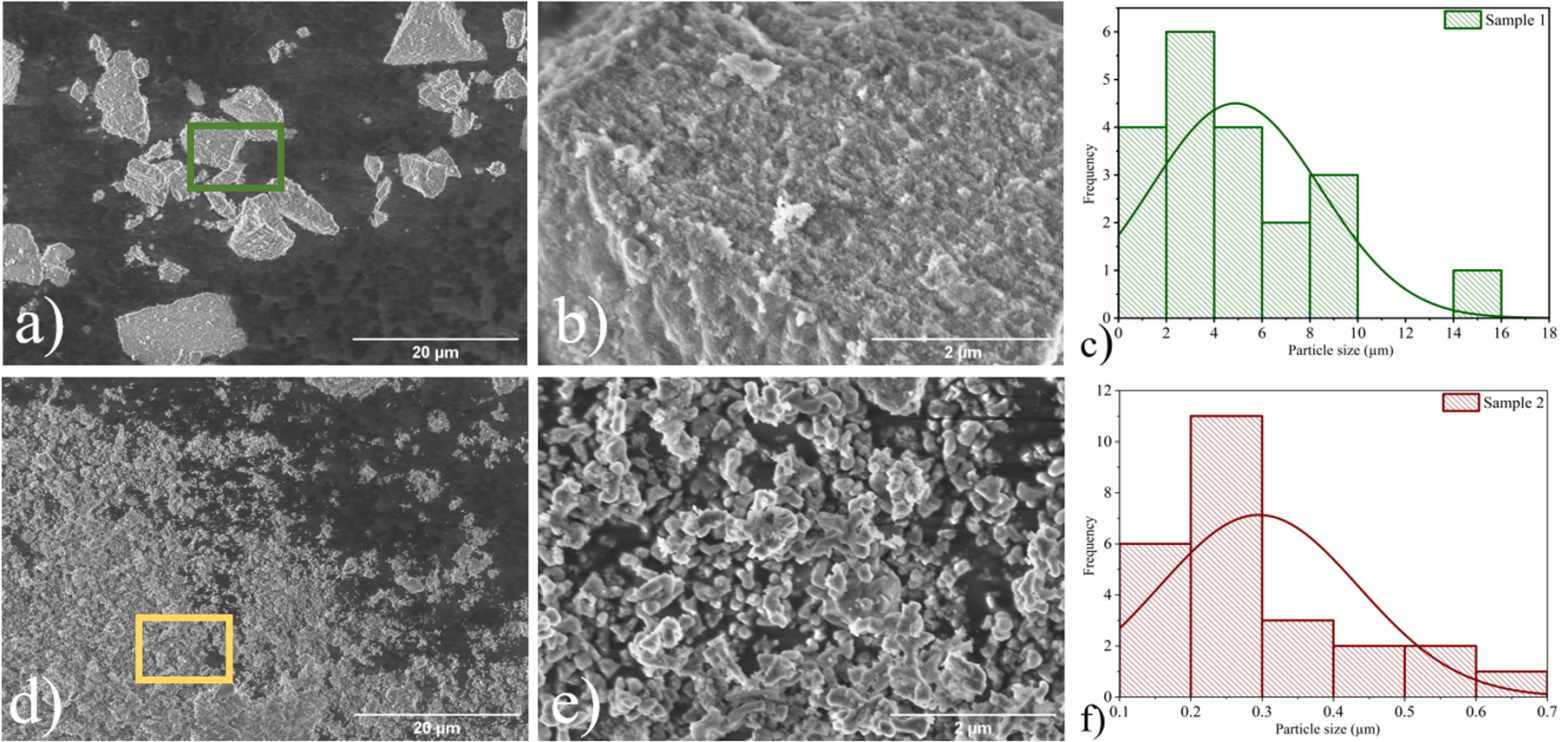
Scanning electron microscopy images of (a) sample 1 (low magnification, SE detector), (b) sample 1 (high magnification, SE detector), (d) sample 2 (low magnification, SE detector), and (e) sample 2 (high magnification, SE detector). Size distribution histograms of (c) sample 1 and (f) sample 2.

The morphology of sample 1 (Figure 3a and Figure 3b) is characterized by large agglomerates with a bed structure and grooves on their surface, with an average agglomerate length of 4.9 μm. Conversely, the morphology of sample 2 is composed of irregular lumps with an average size of 0.29 μm. This type of morphology is characteristic of α-Fe_2_O_3_ nanoparticles [21]. The same morphology was observed for concentrations of 5 and 20 wt % of coloring ions (Figure S2, Supporting Information File 1).

### X-ray photoelectron spectroscopy (XPS)

**Figure 4:**
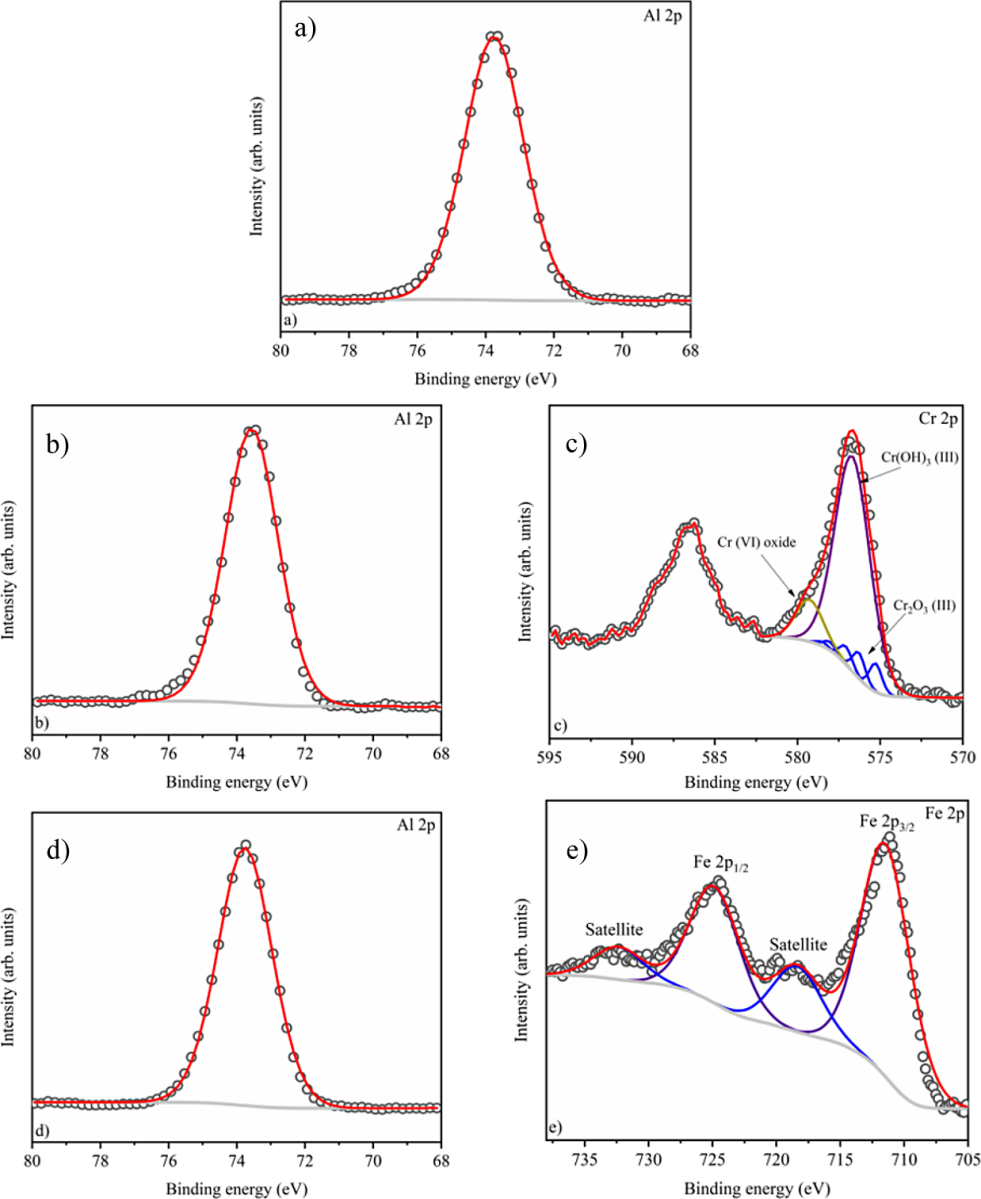
XPS core level spectra. (a) Al 2p of alumina, (b) Al 2p of sample 1, (c) Cr 2p of sample 1, (d) Al 2p of sample 2, and (e) Fe 2p of sample 2. The spectra confirm the oxidation state 3+ in all samples (Al^3+^, Cr^3+^, and Fe^3+^).

The elemental composition of the samples evaluated by the analysis of XPS spectra is shown in Table 2. The relative amount of the coloring ions evaluated by XPS is approximately 12 wt % in both samples. The XPS spectra recorded in the Al 2p core level region (Figure 4a,b,d) show a peak fitted with one component centered at 73.6 eV. This component indicates the presence of Al_2_O_3_ in all samples [22–23], confirming what was observed in XRD. The Cr 2p spectrum (Figure 4c) shows peaks centered at 576.7 and 586.8 eV, which were assigned to Cr 2p_3/2_ and Cr 2p_1/2_, respectively. The fitting analysis of the Cr 2p_3/2_ peak suggests the presence of Cr^6+^ in CrO_3_ (component at 579.3 eV) and Cr^3+^ in chromium hydroxide (Cr(OH)_3_) (component at 576.8 eV) and Cr^3+^ in Cr_2_O_3_ [24]. The characteristic multicomponent of Cr^3+^ (578.7, 578.1, 577.2, 576.3, and 575.3 eV) [24] is caused by the coupling between the unpaired electrons in the core and the unpaired electrons in the outer shell. This can create a series of end states that will be seen in the photoelectron spectrum as a multipeak envelope [25–26]. The Fe 2p spectrum (Figure 4e) shows the characteristic doublet of Fe 2p_1/2_ and Fe 2p_3/2_ at binding energies of 725.1 and 711.1 eV, respectively. The prominent peaks of Fe 2p are accompanied by characteristic Fe^3+^ satellites at higher binding energies (shifted by ca. 9 eV from Fe 2p_3/2_) [27], similar to that reported in [28].

**Table 2:** Composition of alumina, sample 1, and sample 2 samples determined by XPS.

Sample	wt %			

	Al	O	Cr	Fe

alumina	44.9	55.1	—	—
sample 1	38.2	49.4	12.4	—
sample 2	37.3	50.5	—	12.2

### Electronic spectroscopy

**Figure 5:**
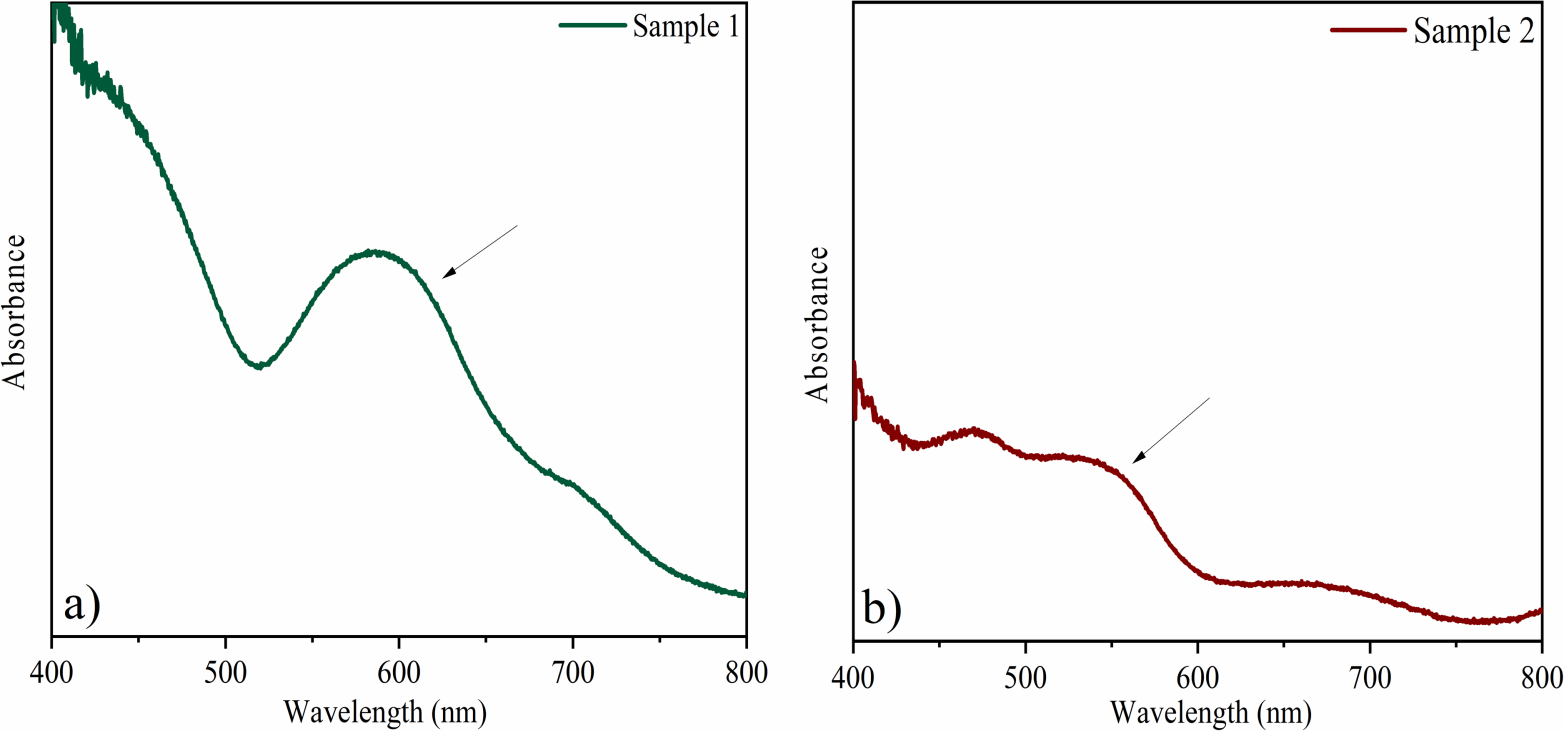
UV–vis absorption spectra of (a) sample 1 and (b) sample 2, both confirming the oxidation state 3+ of the coloring ions.

In the UV–vis absorption spectrum of sample 1 (Figure 5a), the prominent band with a maximum centered at 588 nm can be attributed to the ^4^A_2_→^4^T_2_ transition of Cr^3+^, yielding the green color [1,29]. It suggests a corundum structure, corroborating what was observed in X-ray diffractometry. In the spectrum of sample 2 (Figure 5b), two bands are observed at approximately 480 and 550 nm. These can be related to the transitions ^6^A_1_(^6^S)–^6^A_1_(^6^S)→^4^T_1_(^4^G)–^4^T_1_(G), responsible for the coloring of the hematite phase and confirming the phase indexed in X-ray diffractogram [1,30]. It has been observed that the bands tend to become more prominent as the amount of coloring ions increases for both iron and chromium (see Supporting Information File 1, Figure S3).

### Colorimetry

**Figure 6:**
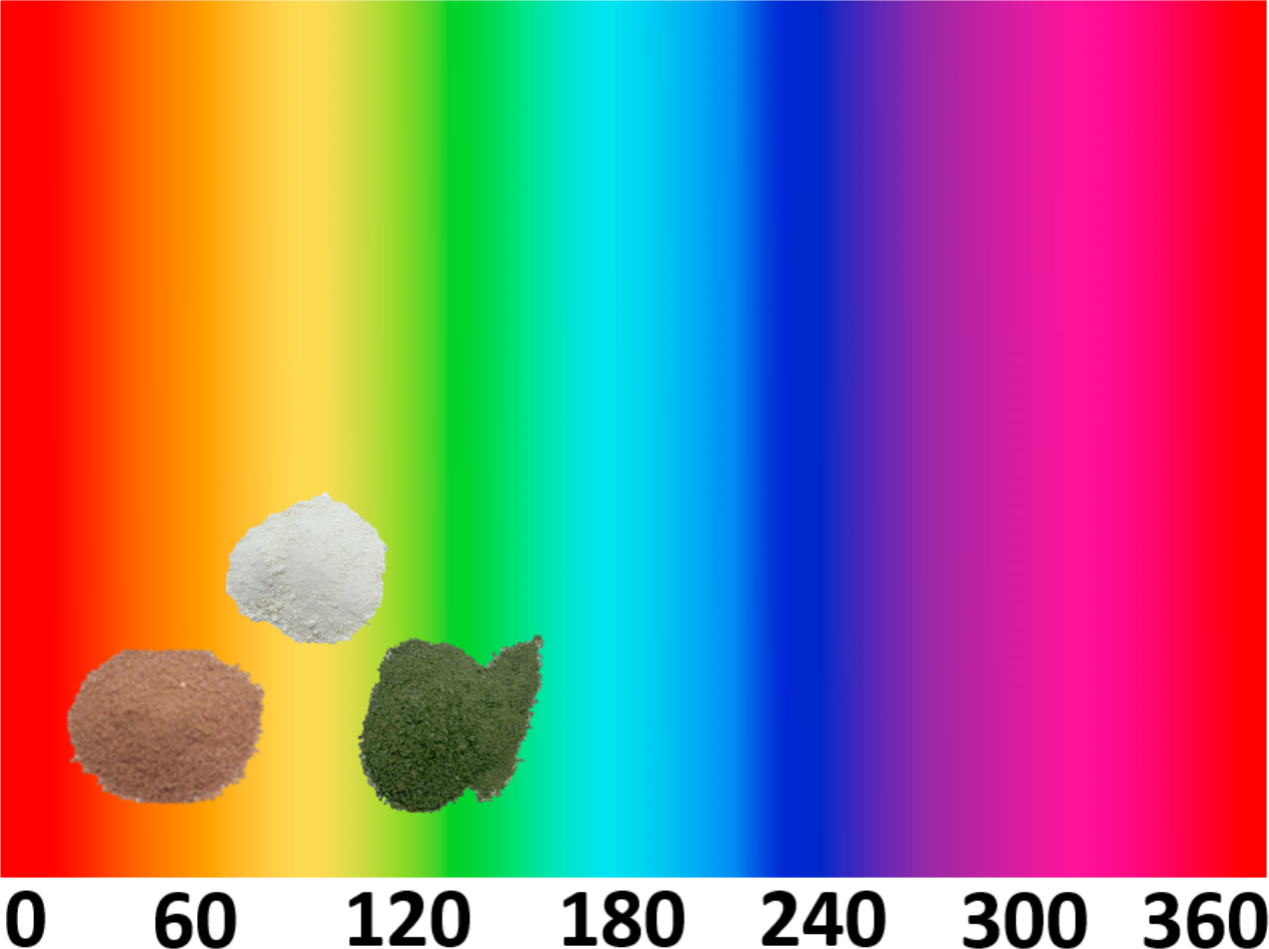
Distribution of samples according to the hue value (h*): alumina (87.20), sample 1 (80.65), and sample 2 (43.44).

Regarding the colorimetric parameters (Table 3), it was observed that the pristine alumina sample is the most luminous (79.75) with the lowest chromaticity (C*). The addition of coloring ions yielded a very strong color difference in the samples in relation to the alumina sample, varying the chromatic parameters and modifying the color [1]. Sample 1 is in the green/yellow quadrant (−a*/+b*), while sample 2 with positive a* and b* values is in the red/yellow quadrant. This is confirmed by hue values (h*) of 80.65 for sample 1 and of 43.44 for sample 2 [31] (Figure 6). The same behavior is observed for the samples with 5 and 20 wt % of coloring ions, with very strong differences when compared to each other (Table S1, Supporting Information File 1).

**Table 3:** Colorimetric parameters of alumina, sample 1, and sample 2.

sample	colorimetric parameters						

	L*	a*	b*	C*	h*	Δ*E*	photo

	alumina	79.75	0.46	9.41	9.42	87.20	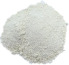
sample 1	35.70	−2.13	12.93	13.11	80.65	44.22	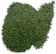
sample 2	33.35	10.29	9.74	14.17	43.44	47.43	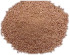

### Reflectance spectroscopy

**Figure 7:**
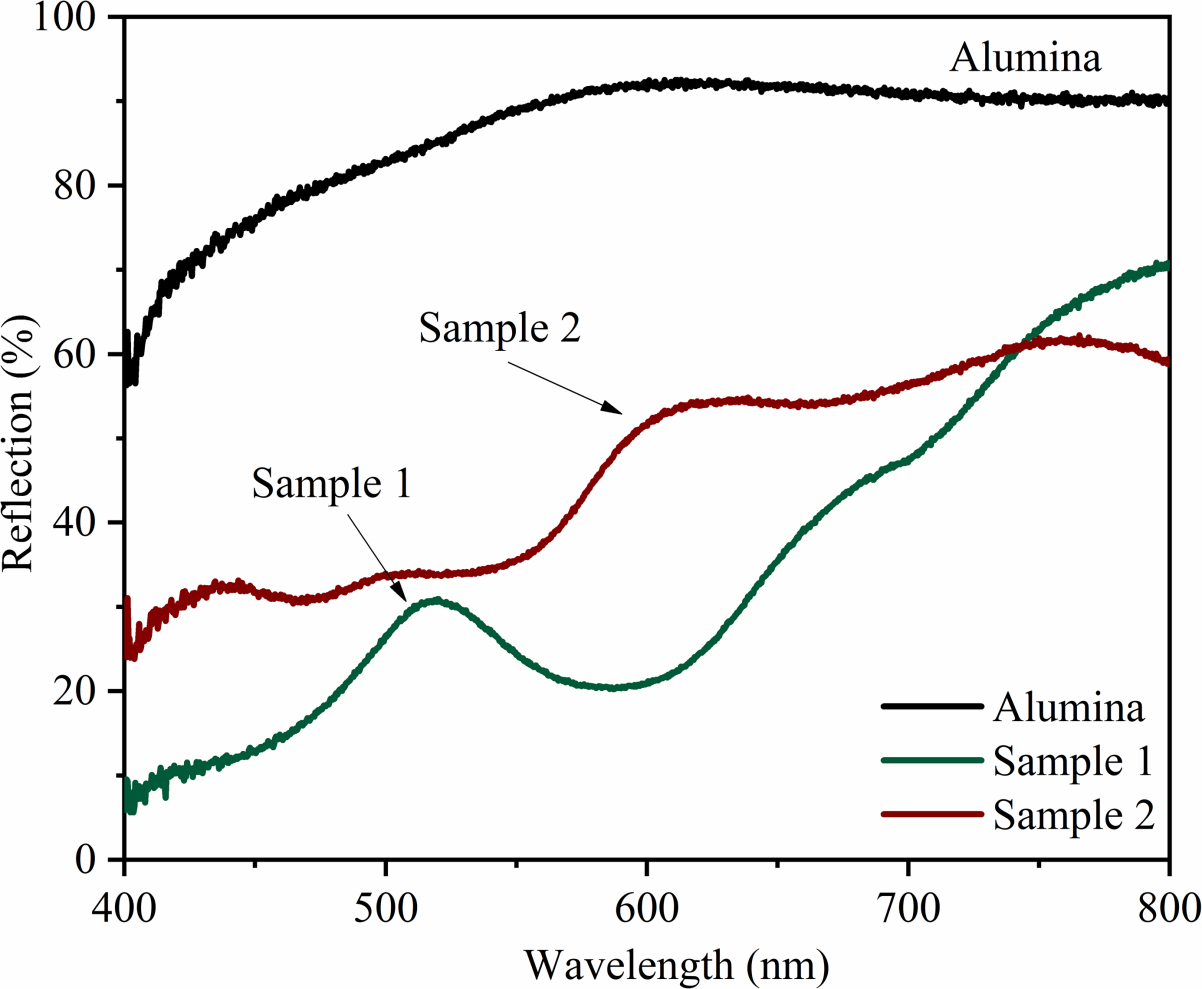
Reflectance spectra in the visible region. Black line: alumina, green line: sample 1, and red line: sample 2, corroborating the colorimetric analysis.

The alumina sample (Figure 7) shows a high percentage of reflectance (90%) from 500 to 800 nm, verifying the high luminosity observed in the colorimetric analysis. Furthermore, the yellowish hue of the pristine pigment was confirmed by the broad band starting at ca. 500 nm. The percentage of reflectance of sample 1 (Figure 7, green line) is consistent with the observed L* value (35.70), indicating low luminance and, consequently, low reflection. The band at approximately 510 nm confirms the yellowish-green color of this pigment. Similarly, the low reflectance of sample 2 (Figure 7, red line) is related to the low luminosity (L* = 33.35) of this sample. The reddish hue color in this pigment was confirmed by the appearance of the broad band from 600 nm. Reflection bands become more pronounced and defined with increasing colorant ion concentration (Figure S4, Supporting Information File 1).

### Pigment application

#### Colorimetry

The colorimetric parameters of the samples after dispersion in commercial white paint and application on plaster blocks are shown in Table 4. It was observed that the chromatic parameters (a* and b*) and the chroma (C*) decreased compared to the powder form, and the luminosity (L*) increased. This can be associated with the white matrix in the paint, which increases the luminosity and decreases the color saturation of the pigments [1]. The hue value (h*) of sample 1 pigment increased from 80.65 (in powder form) to 107.91 after the dispersion in paint, remaining in the green region. Sample 2 remained in the red area.

**Table 4:** Colorimetric parameters of the pigments after dispersion in commercial white paint and application.

Sample	Colorimetric parameters						
	
	L*	a*	b*	C*	h*	Δ*E*	photo
	
white paint	95.83	−0.51	1.47	1.47	109.16	–	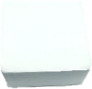
alumina	93.70	−0.49	2.43	2.47	101.38	2.33	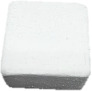
sample 1	86.91	−1.05	3.25	3.42	107.91	9.15	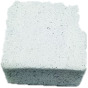
sample 2	78.85	9.03	7.70	11.87	40.45	20.45	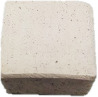

#### Color stability

**Figure 8:**
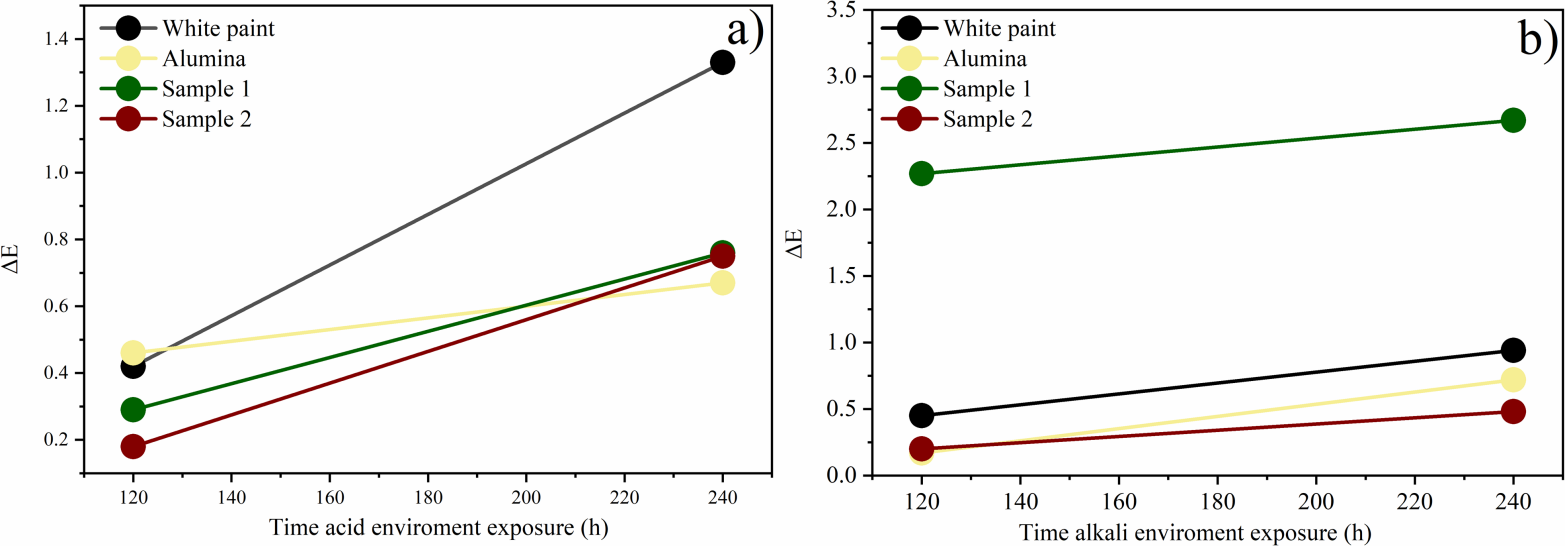
Color stability. Total color difference (Δ*E*) as a function of the exposure time to different environments: (a) acid environment and (b) alkaline environment.

Color stability was measured by colorimetry before the test and after 120 and 240 h of direct exposure to acid and alkaline environments (Table 5). In general, it was observed that the total color variation (Δ*E*) increased after 240 h of exposure in both environments. After 240 h in an acid environment, the commercial white paint showed a Δ*E* of 1.33, which is considered a clear difference. With the application of pigments, the total color difference (Δ*E*) decreased, demonstrating that the pigments increase the stability of commercial paint against aggressive environments. Sample 1 with 20 wt % of coloring ions showed a more stable coloration after 240 h in acidic or alkaline environments. In the case of mixed chromium/aluminium oxide, the highest concentration of coloring ions provided the highest color stability (Table S2, Supporting Information File 1). Similar behavior was observed for samples with Fe. In general, 20 wt % of coloring ions led to better color stability in aggressive environments. This can be associated with a higher stability of the oxides’ crystallographic structure with a greater concentration of coloring ions (Table S2, Supporting Information File 1). Figure 8 shows the Δ*E* values as a function of the exposure time. It indicates that commercial white paint without added pigments exhibits a more significant color variation (Δ*E*) in an acid environment. A work under similar conditions of harsh environments with cobalt and nickel aluminates obtained by the same synthetic route used in this article yielded similar results of color stability [14], with differences varying between weak and very weak. It is possible that the choice of the synthetic route influences the color stability since formation and stability of the structure affect this parameter.

**Table 5:** Colorimetric parameters of alumina, sample 1, and sample 2 applied in white commercial paint after 120 and 240 h exposure to acid and alkaline environment.

Environment	Sample	Colorimetric parameters						

		L*	a*	b*	C*	h*	Δ*E*	photo

acid	white paint – 120 h	95.72	−0.76	1.79	1.96	112.30	0.42	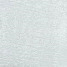
	white paint – 240 h	96.76	0.40	1.18	1.25	71.27	1.33	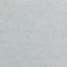

	alumina – 120 h	93.34	−0.50	2.15	2.20	103.10	0.46	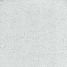
	
	alumina – 240 h	94.26	−0.39	2.08	2.11	100.61	0.67	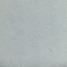

	sample 1 – 120 h	87.12	−1.08	3.12	3.24	109.45	0.29	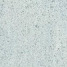
	
	sample 1 – 240 h	86.17	−1.13	3.12	3.27	109.87	0.76	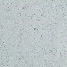

	sample 2 – 120 h	78.48	8.90	7.82	11.85	41.30	0.18	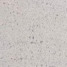
	
	sample 2 – 240 h	79.53	9.23	7.94	12.18	40.70	0.75	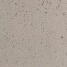

alkaline	white paint – 120 h	95.44	−0.66	1.63	1.76	112.16	0.45	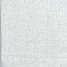
	white paint – 240 h	95.64	0.30	1.03	1.08	73.26	0.94	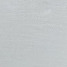

	alumina – 120 h	93.60	−0.38	2.35	2.38	99.18	0.17	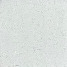
	
	alumina – 240 h	94.35	−0.32	2.18	2.20	98.24	0.72	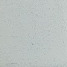

	sample 1 – 120 h	84.90	−1.24	2.21	2.54	119.45	2.27	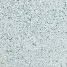
	
	sample 1 – 240 h	84.61	−1.29	1.91	2.30	124.08	2.67	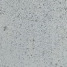

	sample 2 – 120 h	78.90	9.14	7.86	12.06	40.70	0.20	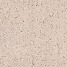
	
	sample 2 – 240 h	78.83	9.03	8.18	12.18	42.15	0.48	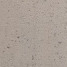

## Conclusion

X-ray diffractometry and Raman spectroscopy indicate the formation of corundum-type mixed oxides together with recycled boehmite. The absorbance spectra indicate the presence of 3+ ions in the samples, which are responsible for the colors. The spectra also confirm the type of structure found via XRD and the oxidation state determined from XPS. SEM images show the characteristic morphology of this type of oxides, confirming that microstructured oxides were obtained from recycled aluminium metal.

The colorimetry measurements indicate a yellowish coloration of pure alumina, a greenish coloration after its combination with chromium, and a reddish coloration after the addition of iron, with hue values of 87.20 for alumina, 80.65 for sample 1, and 43.44 for sample 2. The pigments show color stability in acid and alkaline environments with a slight color variation in the studied time range. The stability can be associated with the stability of corundum-type structures.

## Materials and Methods

### Acid digestion of metallic aluminium

Acid digestion using hydrochloric acid (HCl) was used to recycle aluminium can seals. The HCl concentration was 1.1 mol·L^−1^ using a ratio of 1 g metallic aluminium per 100 mL acid solution. The time required to complete the digestion of aluminium metal was about 24 h. The solution containing the Al^3+^ ions had an acid pH value of 0.45 [1].

### Obtaining boehmite (γ-AlO(OH))

The pH value was modified to obtain the boehmite phase by adding sodium hydroxide (NaOH) until pH 8. After precipitation, the oxide hydroxide was vacuum filtered and oven-dried at 70 °C [1,13].

### Boehmite purification

Formation of sodium chloride (NaCl) during the boehmite synthesis is observed. Therefore, it is necessary to purify the obtained boehmite. Purification was performed by washing the synthesized samples with hot water to remove sodium chloride. After this step, the boehmite was filtered and dried at 70 °C [1,13].

### Oxide synthesis

**Figure 9:**
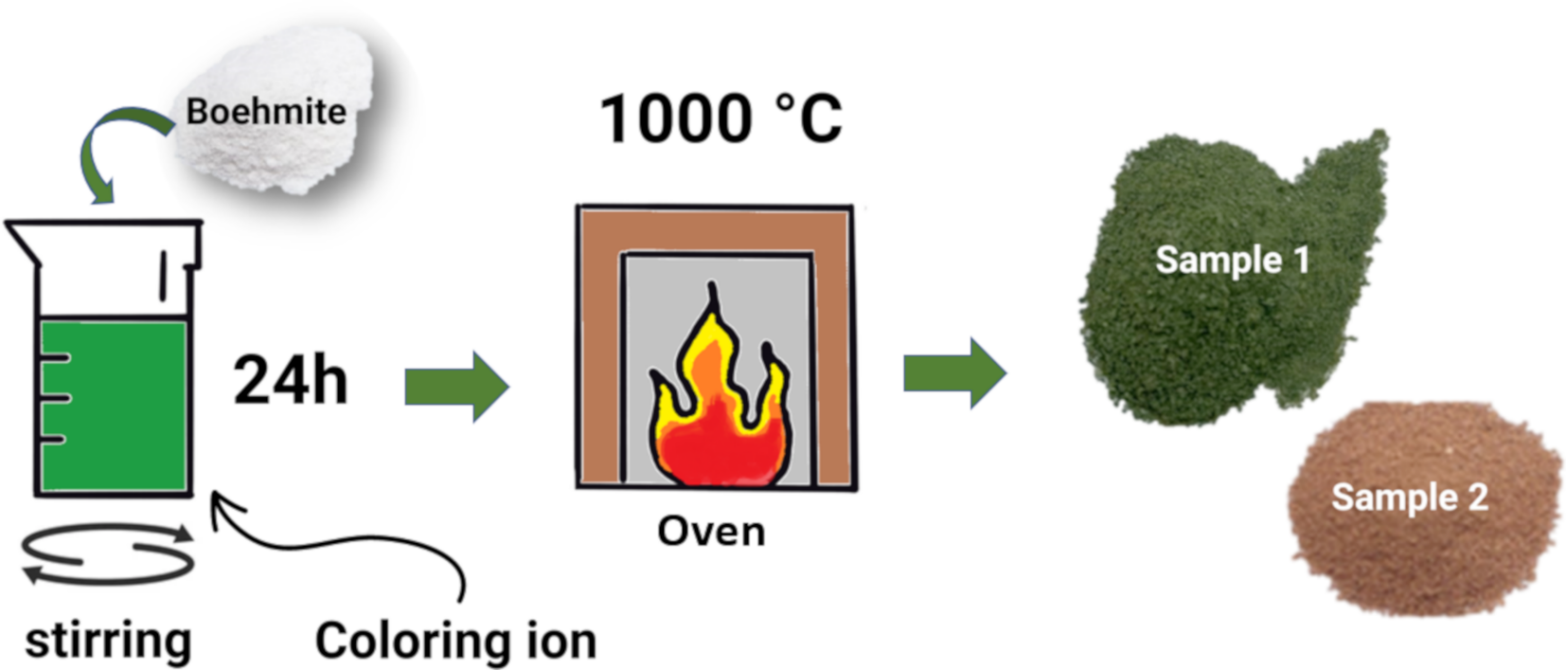
Scheme of the synthetic route used to obtain mixed oxides.

Coloring ions (Cr or Fe) were used for oxide synthesis from boehmite. In the first step, 50 mL of water was added to 10 wt % (in relation to the total mass of boehmite powder (w/w)) of transition metal chloride (CrCl_3_ or FeCl_3_) powder (5 and 20 wt % amounts were also prepared, see Supporting Information File 1). Then, 3 g of boehmite was added and left under constant stirring (600 rpm) for 24 h. Next, this mixture was calcined at 1000 °C, macerated, and stored [14] (Figure 9). The obtained products were labeled “sample 1” for boehmite with chromium atoms and “sample 2” for boehmite with iron atoms.

### Application as pigments

Commercial white paint was used to evaluate the use of sample 1 and sample 2 as pigments. 10 wt % of the colored powder in relation to the total mass of commercial white paint was dispersed in the proportion of 2:1 paint/water [1]. The prepared dispersion was applied on plaster specimens by painting two coats of paint according to the manufacturer’s instructions.

### Color stability

The chemical stability test was carried out in acidic and alkaline environments using desiccators as a controlled experimental environment. A petri dish was placed in each desiccator with hydrochloric acid (HCl) and sodium hydroxide (NaOH), both 1 mol·L^−1^, to yield environments with aggressive vapors [15]. The plaster blocks were placed in the desiccators, which were then sealed. Colorimetric measurements were performed before the test and after 120 and 240 h to determine the colorimetric stability of the aluminates applied as synthetic inorganic pigments [14].

### Characterization

The samples were characterized by powder X-ray diffraction (XRD) carried out in a Bruker D2 Phaser Diffractometer (Berlin, Germany) with Cu Kα emission (λ = 1.5418 Å) and equipped with a LynxEye high-performance detector, with a power of 300 W. A scanning electron microscope Hitachi SU8020 SEM (Tokyo, Japan) was used to obtain morphology information. The oxidation state and composition of the chemical elements at the surface were evaluated by X-ray photoelectron spectroscopy (XPS) (Versaprobe PHI 5000, Physical Electronics, Chanhassen, MN, USA), equipped with a monochromatic Al Kα X-ray source. The spectra were analyzed using the CASA-XPS software. The binding energy of the XPS spectra was calibrated using the C 1s peak at 284.6 eV [32]. Multipack version 9.8 software (ULVAC-PHI, 2017, Chigasaki, Japan) was used to evaluate the relative composition of the elements. Raman spectra were recorded using a micro-Raman system (Senterra Bruker Optik GmbH, Massachusetts, USA), λ = 532 nm with a laser power of 10 mW. The visible spectra were obtained using an Ocean Optics spectrophotometer (USB 2000) (Florida, USA), equipped with optical fiber, tungsten halogen source, and silicon (350–720 nm) and germanium (720–1050 nm) detectors. The colorimetric analysis was performed on the pigments in the form of powder, and after application on the plaster specimens using a portable colorimeter (3nh, model NR60CP, with a D65 light source (Shenzhen, China).

## Supporting Information

File 1 Additional experimental data.
XRD and SEM of the samples, absorption spectra, reflectance spectra, and colorimetric parameters of the oxides and samples.
