# Supplementary material for: Mixed oxides with corundum-type structure obtained from recycling can seals as paint pigments: color stability
Source: Beilstein J Nanotechnol. 2023 Apr 5;14:467–77. doi: 10.3762/bjnano.14.37 (PMC10116409; doi:10.3762/bjnano.14.37)
Supplement: File 1 — Additional experimental data. XRD and SEM of the samples, absorption spectra, reflectance spectra, and colorimetric parameters of the oxides and samples. [file Beilstein_J_Nanotechnol-14-467-s001.pdf]

BEILSTEIN JOURNAL OF NANOTECHNOLOGY

Supporting Information

for

Mixed oxides with corundum-type structure obtained from recycling can seals as paint pigments: color stability

Dienifer F. L. Horsth, Julia de O. Primo, Nayara Balaba, Fauze J. Anaissi and Carla Bittencourt

Beilstein J. Nanotechnol. **2023,** *14,* 467–477. doi:10.3762/bjnano.14.37

Additional experimental data.

XRD and SEM of the samples, absorption spectra, reflectance spectra, and colorimetric parameters of the oxides and samples

License and Terms: This is a supporting information file under the terms of the Creative Commons Attribution License (https://creativecommons.org/licenses/by/4.0). Please note that the reuse, redistribution and reproduction in particular requires that the author(s) and source are credited and that individual graphics may be subject to special legal provisions.
The license is subject to the *Beilstein Journal of Nanotechnology* terms and conditions: (https://www.beilstein-journals.org/bjnano/terms)

X-ray diffractometry (XRD)

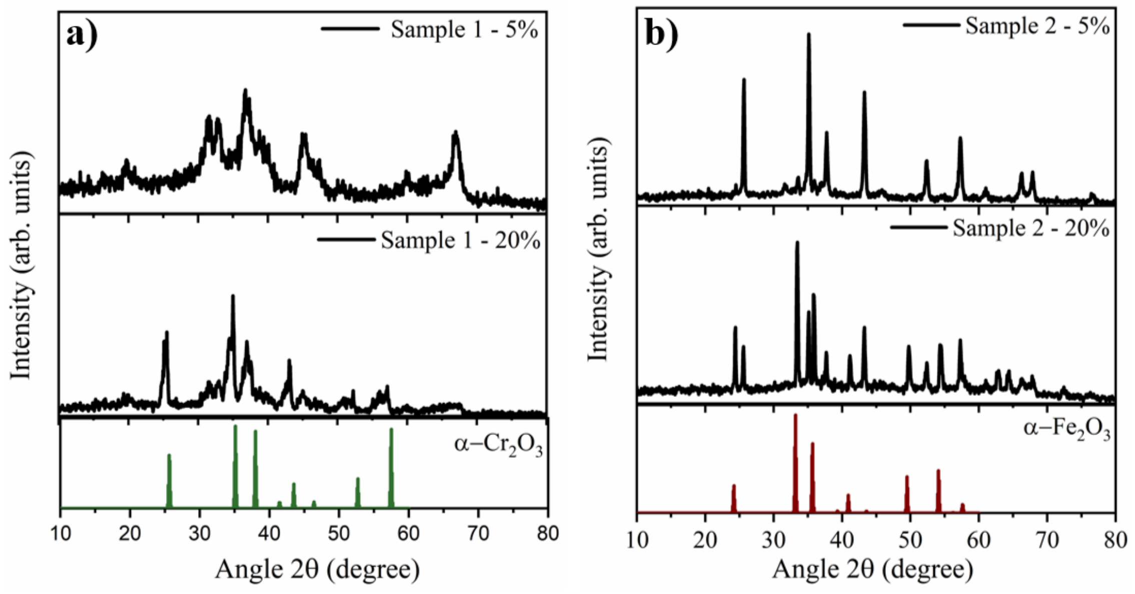

Figure S1: XRD of (a) sample 1 - 5% and sample 1 - 20%, showing α-Cr_2O_3 patterns, and (b) sample 2 - 5% and sample 2 - 20%, showing α-Fe_2O_3 patterns. Both structures are corundum-type with the presence of aluminum oxide.

Scanning electron microscopy (SEM)

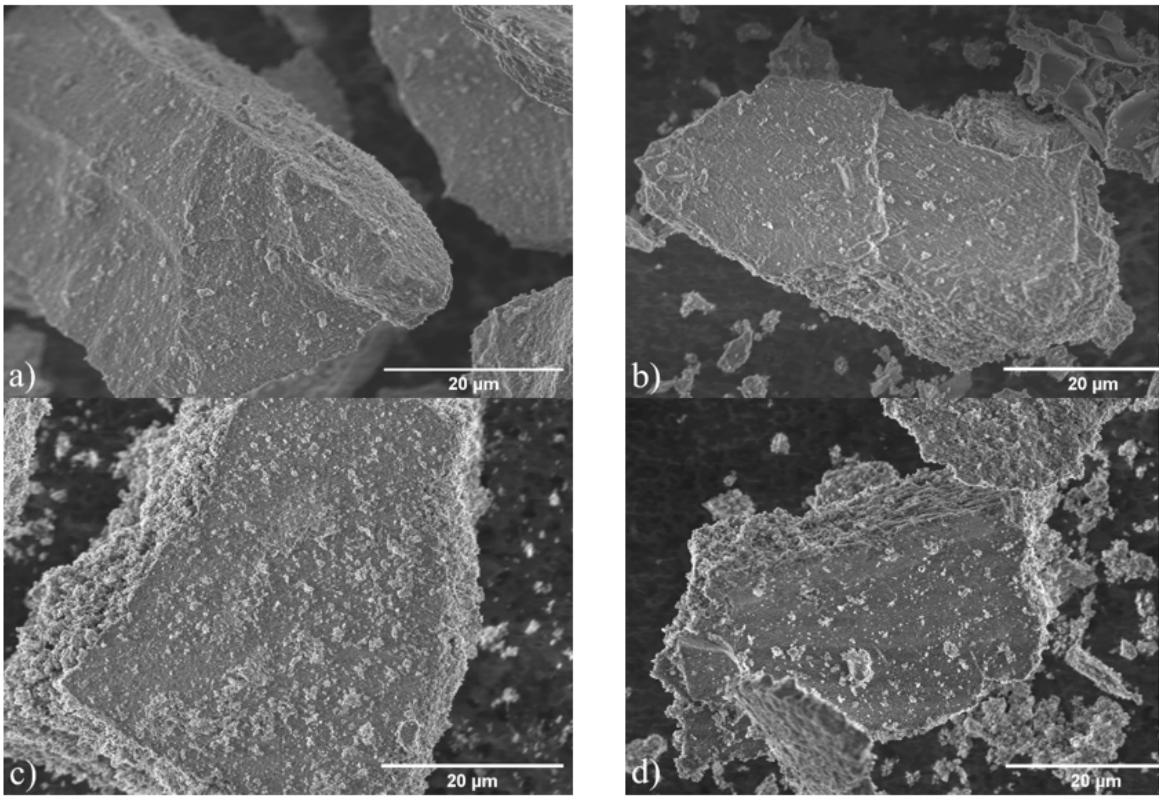

Figure S2: SEM of (a) sample 1 - 5%, (b) sample 1 - 20%, (c) sample 2 - 5%, and (d) sample 2 - 20%. All samples presented large particles with a bed structure and grooves on their surface. Samples 2 - 5% and 2 - 20% are composed of small particles, characteristics of α-Fe_2O_3.

UV–vis absorbance

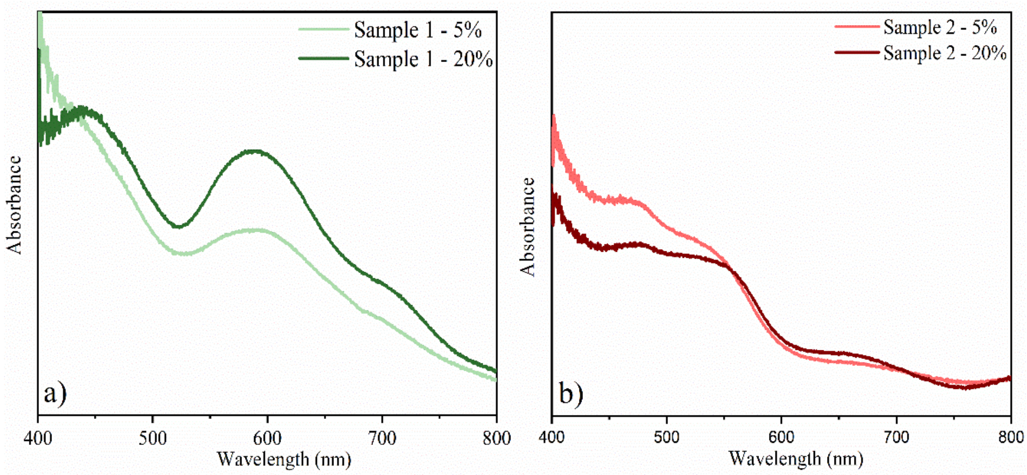

Figure S3: Absorption spectra of (a) sample 1 - 5% and sample 1 - 20% and (b) sample 2 - 5% and sample 2 - 20%. Both spectra show transitions observed in an octahedral structure verifying the XRD results.

UV–vis reflectance:

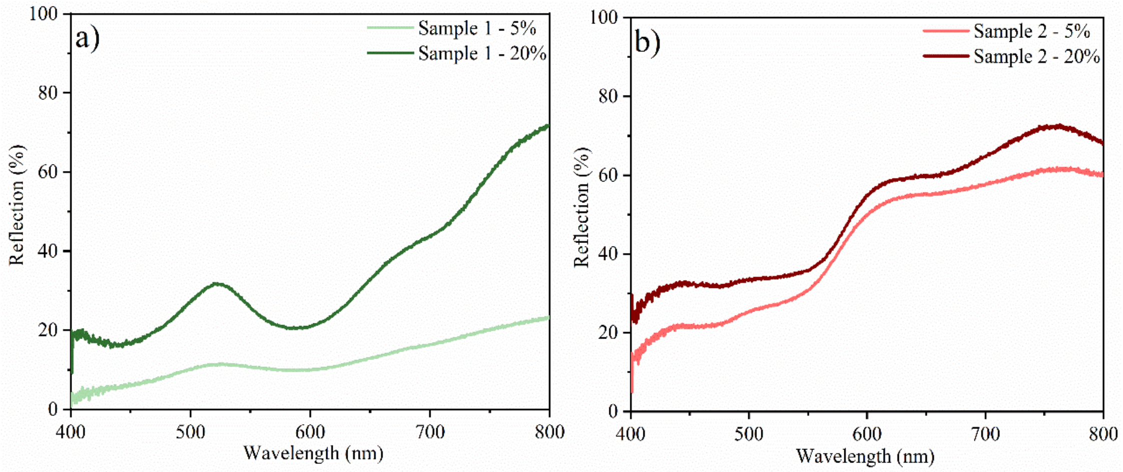

Figure S4: Reflectance spectra of (a) sample 1 - 5% and sample 1 - 20% (the band centered at 523 nm verifies the hue of these pigments) and (b) sample 2 - 5% and sample 2 - 20% (the band centered at 618 nm verifies the hue color of these pigments).

Colorimetry

Table S1: Colorimetric parameters of the oxides.

Sample	Colorimetric Parameters						
	L*	***a****	***b****	***C****	***h****	**Δ*E***	**photo**
alumina	74.34	−0.71	9.48	9.51	94.26	—	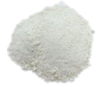
sample 1 - 5%	38.75	0.49	17.73	17.73	88.43	11.24	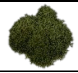
sample 1 - 20%	32.53	−0.35	8.40	8.41	92.36		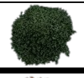
sample 2 - 5%	44.84	16.01	20.90	26.33	52.55	16.76	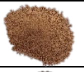
sample 2 - 20%	33.21	11.47	9.72	15.03	40.27		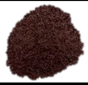

The total color difference (Δ*E*) between similar samples shows a strong color difference caused by the amount of coloring ions.

Color Stability

Table S2: Colorimetric parameters of the samples sample 1 - 5%, sample 1 - 20%, sample 2 - 5%, and sample 2 - 20% applied in white commercial paint after 240 hours in acid and alkali environment exposure.

Environ-ment	Sample	Colorimetric Parameters						
		*L**	*a**	*b**	*C**	*h**	Δ*E*	photo
acid	white paint – 240 h	96.76	0.40	1.18	1.25	71.27	1.33	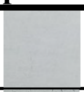
	sample 1 - 5% – 240 h	90.45	−0.74	2.74	2.84	105.12	0.71	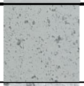
	sample 1 - 20% – 240 h	82.71	−1.80	1.84	2.57	134.31	0.51	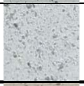
	sample 2 - 5% – 240 h	86.83	6.94	8.08	10.65	49.37	1.09	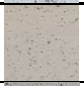
	sample 2 - 20% – 240 h	79.95	5.52	2.42	6.03	23.71	0.92	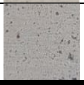
alkaline	white paint – 240 h	95.64	0.30	1.03	1.08	73.26	0.94	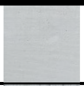
	sample 1 - 5% – 240 h	89.40	−0.87	3.37	3.49	104.50	1.27	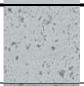
	sample 1 - 20% – 240 h	82.52	−1.93	1.29	2.32	146.26	0.25	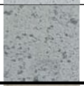
	sample 2 - 5% – 240 h	85.97	7.29	8.93	11.53	50.83	0.91	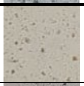
	sample 2 - 20% – 240 h	79.06	5.22	2.45	5.77	25.13	0.16	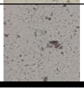

The total color difference (Δ*E*) indicates that the higher the percentage of coloring ions in the oxide, the more stable its color.
